# LncRNA Subcellular Localization Across Diverse Cell Lines: An Exploration Using Deep Learning with Inexact *q*-mers

**DOI:** 10.3390/ncrna11040049

**Published:** 2025-06-25

**Authors:** Weijun Yi, Jason R. Miller, Gangqing Hu, Donald A. Adjeroh

**Affiliations:** 1 Computer Science and Electrical Engineering, West Virginia University, Morgantown, WV 26506, USA; wy0003@mix.wvu.edu; 2 Computer Science and Information Technology, Hood College, Frederick, MD 21701, USA; jmiller@hood.edu; 3 Microbiology, Immunology, and Cell Biology, WVU School of Medicine, Morgantown, WV 26506, USA; michael.hu@hsc.wvu.edu

**Keywords:** lncRNA localization, 1D-CNN, inexact *q*-mers, switching genes

## Abstract

**Background:** Long non-coding Ribonucleic Acids (lncRNAs) can be localized to different cellular compartments, such as the nuclear and the cytoplasmic regions. Their biological functions are influenced by the region of the cell where they are located. Compared to the vast number of lncRNAs, only a relatively small proportion have annotations regarding their subcellular localization. It would be helpful if those few annotated lncRNAs could be leveraged to develop predictive models for localization of other lncRNAs. **Methods:** Conventional computational methods use *q*-mer profiles from lncRNA sequences and train machine learning models such as support vector machines and logistic regression with the profiles. These methods focus on the exact *q*-mer. Given possible sequence mutations and other uncertainties in genomic sequences and their role in biological function, a consideration of these variabilities might improve our ability to model lncRNAs and their localization. Thus, we build on inexact *q*-mers and use machine learning/deep learning techniques to study three specific problems in lncRNA subcellular localization, namely, prediction of lncRNA localization using inexact *q*-mers, the issue of whether lncRNA localization is cell-type-specific, and the notion of switching (lncRNA) genes. **Results:** We performed our analysis using data on lncRNA localization across 15 cell lines. Our results showed that using inexact *q*-mers (with *q* = 6) can improve the lncRNA localization prediction performance compared to using exact *q*-mers. Further, we showed that lncRNA localization, in general, is not cell-line-specific. We also identified a category of LncRNAs which switch cellular compartments between different cell lines (we call them switching lncRNAs). These switching lncRNAs complicate the problem of predicting lncRNA localization using machine learning models, showing that lncRNA localization is still a major challenge.

## 1. Introduction

Non-coding RNAs (ncRNAs) and protein-coding genes are two constituent parts of the human genome [1]. Conventionally, non-coding RNAs can be divided into small ncRNAs with sequence length less than 200 nucleotides and long non-coding RNAs (lncRNAs) with sequence length greater than or equal to 200 nucleotides [2]. The lncRNA family has grown rapidly since its discovery in the early 1990s, with over 270,000 lncRNA transcripts identified in humans [3]. Unlike the protein-coding genes, which are functional units of heredity [4], non-coding RNAs (ncRNAs) were once deemed non-functional. They were perceived as the product of spurious transcription [5]. However, high-throughput sequencing technologies [6] have shed more light on these ncRNA. Various research groups have shown that ncRNAs, especially lncRNAs, exhibit biological functions including chromatin modification, cell cycling, protein transcription, and translation [7,8]. They have also been associated with different diseases, including cancer, autism, Alzheimer’s disease, and others [9,10,11]. The critical role of lncRNAs in complex diseases is underlined by the existence of over 10,500 experimentally supported lncRNA–disease associations, for instance as recorded in LncRNADisease [12], a popular database of lncRNA-associated diseases. Similarly, several recent reports have identified miRNA–disease associations using different models [13,14,15].

Similar to proteins, the subcellular localization of lncRNAs is linked to their function in the cell [16]. Therefore, understanding the subcellular localization of lncRNAs and their dynamic changes can help to explain the function of newly discovered lncRNAs [17]. RNALocate v2.0 [18], a database constructed in 2016 and updated in 2021, contains 9594 lncRNA entries, in which 6731 unique genes are localized in the nucleus, cytoplasm, ribosome, exosome, nucleoplasm, chromatin, cytosol, endoplasmic reticulum, plasma membrane, etc. LncATLAS [19], another database for the subcellular localization of lncRNAs, was introduced in 2017. They considered the cytoplasmic/nuclear relative concentration index (CN-RCI), which is derived from GENCODE (Ensembl) RNA-Seq measurements, as a key measure of localization. Essentially, the CN-RCI (defined in log2 units) measures the relative concentration of an RNA sequence in the cytoplasm compared to the nucleus. A higher positive CN-RCI value of the lncRNA (i.e., log(CN-RCI) ≥ 0 in log2 units) suggests that the lncRNA is predominantly localized in the cytoplasmic compartment, while a lower negative CN-RCI value (log(CN-RCI) < 0) indicates that it is localized in the nuclear compartment. LncATLAS provided CR-RCI values for 6768 GENCODE-annotated lncRNAs across 15 cell lines.

Recent studies have explored the use of machine learning/deep learning techniques in identifying lncRNAs [20,21]. Computational approaches have been developed to predict lncRNA subcellular localization from known subcellular localization datasets. These studies extract short nucleotide segments (*q*-mers or *q*-grams) from lncRNA sequences and then apply machine learning models, such as Random Forest (RF), support vector machines (SVMs), or deep neural network models to make predictions on the localization [22,23,24,25]. Traditional computational methods have focused on exact *q*-mers.

In this work, we use the notion of inexact *q*-mers (that is, *q*-mers with mismatch, see [26]) to explore the problem of lncRNA localization under a deep learning framework. Methods for matching with inexact *q*-mers, or approximate *q*-mer/*q*-grams, are well established in string pattern matching [27,28,29,30]. To our knowledge, using inexact *q*-mers in deep learning prediction of lncRNA localization was first introduced in [26], which was a shorter version of this work. DNA sequences are known to undergo changes due to random mutations or errors that occur during DNA replication. Such mutations have been associated with the development of various diseases, including Parkinson’s disease, cancer, genetic disorders, Alzheimer’s disease, and various other complex diseases [31,32,33,34]. Mutations or errors in the lncRNA sequence could also be introduced at the time of sequencing [35,36]. Applying *q*-mer alignment with mismatches is useful for identifying similarities and differences between two DNA sequences, even if they are not identical. By allowing for a certain number of mismatches, *q*-mer alignment can be more sensitive in detecting related sequences that may have undergone minor changes due to mutations or sequencing errors. The number of allowed mismatches in *q*-mer alignment can be adjusted depending on the application and expected level of similarity between the sequences being compared. Further, we analyzed the lncATLAS localization data to elucidate the question of cell specificity with respect to lncRNA localization. The results show that accurate classification of lncRNA subcellular localization using only *q*-mer-based information is still a major challenge.

## 2. Background and Related Work

### 2.1. Subcellular Localization

**Figure 1:**
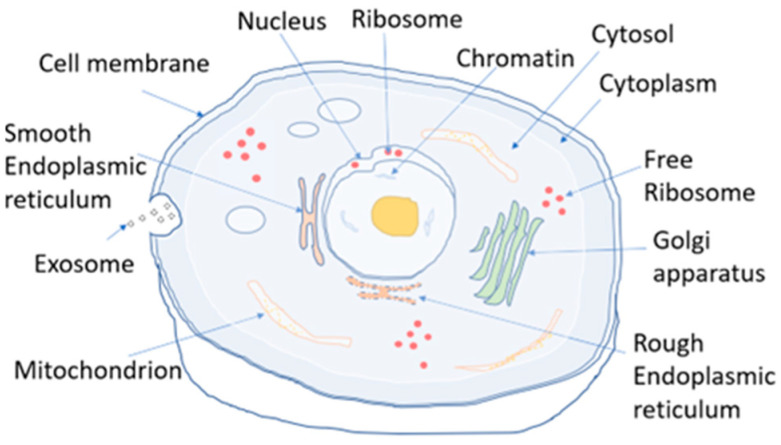
The structure of an animal cell. The key target lncRNA localizations in most datasets are nucleus, cytosol, and cytoplasm.

RNAs play crucial roles in cellular processes, including translating genetic information, regulating gene activity, and involvement in cellular differentiation [37]. These functions are significantly influenced by the RNA localization in the cell [17,38,39]. The cell of eukaryotic organisms can be divided into functionally distinct membrane-bound compartments [23] (See Figure 1), which are linked with different biological processes [40]. To understand the function of RNA, we need to understand its subcellular localization. Experimental methods, such as FISH, used to study RNA subcellular localization, require knowledge of molecular chemistry and specialized instruments and techniques [20,41,42].

RNAs are typically divided into coding and non-coding types based on their coding potential [43]. Coding RNAs encode protein. Non-coding RNAs act as cellular regulators without encoding proteins. Unlike the coding RNAs, which have been studied widely, long non-coding RNAs (lncRNAs) are more difficult to explore given their low expression levels [44]. Thus, using information from known datasets to predict the subcellular localization of lncRNAs has become an important endeavor, but still remains a significant challenge [45]. There are existing databases [19,22,23] which annotate lncRNAs with their subcellular localizations, such as cytoplasm, nucleus, ribosome, exosome, etc. Therefore, we can treat the prediction of subcellular localization as a classification problem. For coding RNAs, there are many predictors of protein localization, which have been developed since the 1990s [46,47,48,49,50]. Many of these use computational approaches, such as artificial neural networks, or support vector machines (SVMs), for example, in [50]. However, in contrast to protein-coding RNAs, relatively fewer methods have been proposed for predicting lncRNA subcellular localization.

### 2.2. Prior Computational Approaches

General computational methods predict the localization of lncRNAs by extracting *q*-mer features from the lncRNA sequence. Some of these methods select nucleotide segments as their basic features. In [51], the pseudo-*k*-tuple nucleotide composition (PseKNC) was used as a discrete model to represent RNA sequences. Under the PseKNC model, *q*-length substrings (*q*-mers) are extracted from the RNA sequence. Each substring can be treated as an RNA motif that contains some biological information. By decomposing the RNA sequence into small segments, it becomes more efficient to analyze compared to analyzing long RNA sequences. This concept was demonstrated in a study by Kirk et al., who utilized profiles based on *q*-mers to investigate the subcellular localization of lncRNAs [52].

In LncLocator [22], Cao et al. created an annotated subcellular localization dataset of lncRNAs from RNALocate [53]. The dataset contained 612 lncRNAs localized to five locations in the cell, namely, the nucleus, cytoplasm, cytosol, ribosome, and exosome. They extracted *q*-mer segments (*q* = 4,5,6) from the lncRNA sequences. Given the low discrimination ability of very short segments, they fed 4-mer features into a stacked autoencoder model to create a high-level feature representation of the sequence. They tested their data and approach in various scenarios. The overall accuracy was 59.8% on the five-class dataset. Their model was trained and validated on a small dataset with uneven classes, 301 cytoplasm and 25 exosome, which limited its generalization capabilities to new datasets.

Another method, iLoc-lncRNA [23], predicts lncRNAs’ subcellular localization by feeding octamer sequence features into an SVM model. They build a four-class dataset with 655 lncRNAs based on the RNALocate database [53]. The classes correspond to the following localizations: nucleus, cytoplasm, ribosome, and exosome. First, they extracted 8-mer features from the lncRNA sequences. Then, to reduce dimensionality, redundancy, and possible overfitting, they selected features based on the probability distribution of the 8-mer features. This resulted in 4107 selected 8-mer features from the 4^8^ possibilities. Then, they trained an SVM model with the extracted features. The overall accuracy was 86.72% on the four-class dataset. The model was trained on a relatively small dataset, which may not generalize well to novel datasets. Using a high-dimensional feature space (65,536 8-mers) increases computational cost. While feature selection methods were employed to mitigate this, the large feature set could introduce noise and affect model performance.

Gudenas et al. [24] built a two-class dataset from the ENCODE project. First, they quantified the lncRNA transcript differences between nuclear and cytosolic, applying a log2 fold change threshold to allocate 8678 lncRNAs to cytosolic (log2 value < 0) and nuclear (log2 value > 2.8), resulting in 4380 for cytosolic and 4298 for nuclear. They then extracted *q*-mer features (*q* = 2,3,4,5) from the lncRNA sequences. Next, they added RNA–protein binding motifs to the feature map and passed these to a deep neural network. They obtained an accuracy of 72.4%. Fan et al., in lncLocPred [54], built a four-class dataset from the RNALocate database [53]. The database contained 396 lncRNAs. They used this dataset as an independent dataset and dataset in iLoc-lncRNA [23] as the benchmark dataset. First, they collected features using *q*-mers (*q* = 5,6,8), a local structure–sequence triplet element, and pseudo-dinucleotide composition. They then trained a logistic regression model using the selected features.

Yuan et al. [25] built a localization dataset from published datasets, such as CeFra-seq, APEX-Seq [55], and lncAtlas [19]. They used the annotation file from GENCODE [56] v30 and removed RNAs that were shown to be inconsistent with multiple localizations across the publicly available datasets. Based on these, they introduced the RNAlight model building on the Light Gradient Boosting Machine [57] to predict RNA subcellular localization by using *q*-mer frequencies. In their study, the mean value of CN-RCI of 13 cell lines were used as a filter. They fixed the thresholds to −2 and 0. LncRNAs with a mean log2 (CN-RCI) < −2 were assigned to nuclear class, while those with a mean log2(CN-RCI) > 0 were placed in the cytoplasmic class. They obtained an overall accuracy of 0.72 on their final test set, which contained 380 lncRNAs.

Jeon et al. [58] developed a tree-based algorithm for cell-specific lncRNA subcellular localization utilizing the lncLocator2 [59] database. Based on their proposed method, they performed cross-model validation and argued that lncRNA localization is cell-specific. That is, the high accuracy prediction of lncRNA subcellular localization only occurred when the training set and test set both came from the same cell line. Thus, a model trained on a given cell line may not perform well when tested on a different cell line. Previously, Zuckerman and Ulitsky [60] developed models for predicting lncRNA subcellular localization (and also mRNA localization) based on several feature types, including information on splicing and gene structure (introns, exons), hexamers (A-rich preference and C-rich preference), gene expression, histone modification, Pol2, etc. Using these attributes, they developed models based on Random Forest and linear regression. They used a threshold of log2(CN-RCI) of ±1 to assign the lncRNAs into cytoplasmic and nuclear lncRNAs. They obtained precision and recall values of about 60%. They suggested that lncRNA localization may not be cell-specific in that the same prediction model can be used for successful prediction on different cell lines. We note that this assertion is in contrast to the more recent suggestion by Jeon et al. [58] above. These contrasting views deserve a more careful analysis.

As can be observed, most of the prior work have excluded lncRNAs with log2(CN-RCI) values ranging within a certain range, typically around 0. For instance, from 0 to 2.8 in [24,50], from −2.0 to 0 in [53], and from −1 to +1 in [53,55]. This middle exclusion problem (see [56] below) can limit the model’s capability to accurately predict lncRNAs that fall within the intermediate CN-RCI range in new datasets. Further, none of the methods accounted for potential sequence variations in lncRNAs due to mutations, which could improve the accuracy and robustness of their predictions.

A recent study [61] introduced a new benchmark on lncRNA subcellular localization. They identified possible data leakage in some published lncRNA subcellular localization studies. They reviewed four existing models. In these models, the lncRNA subcellular localization was decided by the CN-RCI value. They observed that thresholds on CN-RCI values significantly affected the classification accuracy. Thus, they noted a problem they called the “middle exclusion” problem, whereby the two thresholds are used to define cytoplasmic vs. nuclear localization, thus essentially ignoring the middle region. This can be observed in some of the work cited above (e.g., [24,25,54]). By setting 0 as the threshold for cytoplasm/nuclear localization, models achieved only about 60% accuracy. However, excluding the genes with CN-RCI values within the middle range tended to inflate the classification performance, for instance, 72–74% using middle exclusion methods. In the study, the authors did not evaluate the impact of mismatches in *q*-mer alignments, which could potentially improve classification performance.

We will test our methods primarily with the data used in [61]. Table 1 shows the current comparative models on lncRNA subcellular localization that use *q*-mers as their main feature. lncLocator [22] and iLoc-lncRNA [23] extracted the dataset from RNALocate [53], and they considered multiple compartments (five and four subcellular localizations, respectively). The other four methods operate on binary classes (cytoplasmic or nuclear). They used thresholds on the log2 (CN-RCI values) to decide on the subcellular localization. Most of these applied middle exclusion: the lncRNAs were excluded if the log2 value was between the two thresholds. DeepLncRNA [24] extracted the dataset from the ENCODE project. RNAlight [25] and TACOS [58] built the dataset from GENCODE and lncATLAS [19]. They used all the transcripts from the genes and applied some filters to build the dataset. As suggested in [56], in our study, we used only one threshold (0 in our case) to decide on cytoplasmic vs. nuclear localization to avoid the problem of middle exclusion. To reduce the workload, we chose lncRNAs with a length of between 200 and 5000.

## 3. Results

### 3.1. Inexact q-mers Improve Prediction of LncRNA Subcellular Localization

First, we tested our (*q*,*k*)-mismatch models on lncRNAs from the LncATLAS dataset. For this initial experiment, we worked on four selected cell lines, namely, HT1080, A549, NCI.H460, and SK.N.SH (see Section 5). Table 2a shows the validation results using our proposed model with latent features on the four cell lines for three classifiers with different mismatches. We tested with different numbers of latent features (512, 256, 128, 64, and 32) and found 512 latent features to provide the overall best results. In the table, “kmiss oa” denotes overall accuracy when using 6-mer with *k* mismatches; here, *k* is from 0 to 3. While “kmiss auc” denotes the area under the receiver operating characteristic curve when using *k*-miss, *k* is also from 0 to 3. In all cases, the highest accuracy for each classifier was observed when we applied inexact 6-mers (i.e., *k*-mismatch with *k* > 0). Table 2b shows more detailed results using 1DCNN with 512 latent features.

Supplementary Table S1a,b show corresponding results for using SMOTE [62] and class weight during training for data imbalance for the case of using all 4096 features.

The results revealed three key observations: (1) in general, when predicting lncRNA subcellular localization, the 1D-CNN and RF classifiers performed better than MLP in this study, both with all 4096 features and the 512 latent features that were extracted from the 4096 features; (2) working with SMOTE was more effective than weight fitting during training for our lncRNA localization problem; and (3) the (*q*,*k*)-mismatch model worked well for predicting subcellular localization. The highest scores occurred when there was some mismatch (*k >* 0), showing the performance improvement with inexact *q*-mers over traditional exact *q*-mers. In particular, 6-mers with two mismatches usually produced the best performance on our dataset. In the following, we will focus on the (6,2)-mismatch model.

To further evaluate the proposed approach using inexact *q*-mers, we applied 6-mers with up to three mismatches on mRNA transcripts from the LncATLAS dataset, following the same general procedure used for lncRNAs (four cell lines: HT1080, A549, NCI.H460, and SK.N.SH). Table 3a,b show the results for 512 latent features. Supplementary Table S1c shows the results using all 4096 6-mers. Similar to lncRNAs, we applied SMOTE to handle the data imbalance problem. When using 512 latent features, 6-mers with two mismatches achieved the highest accuracy of 67.63%, with the highest AUC of 0.74 (compared with 63.69% accuracy and an AUC of 0.69 using exact 6-mers). Thus, similar to lncRNAs, using inexact *q*-mers (with mismatch) also improved localization prediction performance for mRNAs.

To further evaluate the generality of the proposed approach, we also tested the (*q*,*k*)-mismatch model on the APEX-Seq dataset using the same procedure as above. First, we observed the heavily imbalanced nature of LncRNA data (see datasets in Section 5). With the log fold change (logFC) threshold (log FC ≥ 0.75), APEX-Seq had only 34 LncRNAs (4 cytoplasmic, 30 nuclear). Thus, we could not use this for training the model. Rather, we used these as a test set for the model trained using the LncATLAS dataset and log2(CN-RCI) threshold of 0. The results are shown in Table 4a. Overall, the results are similar, though generally lower than what we obtained when we trained and tested on the LncATLAS dataset (Table 2a), except for the 1DCNN model. Although the accuracy was comparable, the AUC values in Table 4a were relatively lower. We suspect that this disparity in performance may be due to the potential difference in the meaning of the two thresholds used for the two datasets. It is possible that the log fold change threshold (log FC ≥ 0.75) used for APEX-Seq may not necessarily correspond to the CN-RCI threshold (log CN-RCI ≥ 0) used in LncATLAS to define the two classes.

Since we have a larger amount of data on mRNAs from the APEX-Seq dataset, we repeated our prior experiments on mRNA sub-cellular localization. Here, we were able to train and test the models using data from APEX-Seq using the log FC ≥ 0.75 threshold (using one combined dataset, not four cell lines this time). Similar to Table 3a on mRNA results with LncATLAS, Table 4b shows the results for 1DCNN with 512 latent features when applied on the APEX-Seq mRNA dataset. As with Table 3a, the best results (highest OA and AUC) in Table 4b were observed with inexact match, mostly using the (6,1)-mismatch or (6,2)-mismatch models for this dataset. The results from APEX-Seq (Table 4b) are comparable with (though generally lower than) those from LncATLAS (Table 3a).

To further investigate the generality of the approach, we also evaluated the performance of the proposed approaches on the Ribosome lncRNA dataset [63]. Unlike the LncATLAS dataset, this dataset is a non-cellular fractionation dataset, similar to the APEX-Seq dataset [55]. Given the size of the dataset (272 lncRNA genes, 155 nuclear, and 117 cytoplasmic; see Materials and Methods), we were able to both train a model for lncRNA localization and also validate it using the Ribosome dataset. The results are shown in Table 5a. (We note that, for the experiments on the Ribosome lncRNA dataset, we show the results for using the 4096 (6,*k*)-inexact 6-mer features (that is, without the autoencoder). Using the autoencoder generally resulted in a lower performance on this dataset). As can be observed in the table, the results can be compared with those obtained for lncRNA localization using the LncATLAS dataset (Table 2a). Table 5a (results for Ribosome dataset) shows a slightly better AUC, while Table 2a (results for LncATLAS dataset) shows better accuracy.

As a second experiment on the Ribosome lncRNA dataset, we used the entire dataset as a test set for a model trained using the lncATLAS dataset with a log CN-RCI threshold of 0. (This is similar to what we did for the APEX-Seq dataset (Table 4a).) To avoid possible data leakage between training and test sets, for this experiment, we removed the lncRNAs that appeared in the APEX-Seq dataset or in the Ribosome dataset from the LncATLAS dataset before training. The results are shown in Table 5b. Once again, the results with the Ribosome dataset (Table 5b) showed a better AUC, while results on the APEX-Seq dataset showed better accuracy. As was noted in previous experiments, the best results are usually observed using inexact *q*-mers (i.e., with *k >* 0), showing that inexact *q*-mers produce improved results over exact *q*-mer profiles, even with cross-dataset training and testing.

Overall, the results on the APEX-Seq and Ribosome lncRNA datasets are similar, though generally lower than what we obtained when we trained and tested on the LncATLAS dataset (Table 2a,b), except for the 1DCNN model. Although the accuracy was comparable for the LncATLAS (Table 2a) and APEX-Seq dataset (Table 4a), the AUC values in Table 4a (for APEX-Seq) were relatively lower. Similarly, the AUC was comparable between the LncATLAS and Ribosome datasets, while the accuracy with the Ribosome dataset (Table 5a) was lower. We suspect that this disparity in performance may be due to the potential difference in the meaning of the two thresholds used for the two datasets. For instance, it is possible that the log fold change threshold (log FC ≥ 0.75) used for APEX-Seq may not necessarily correspond to the CN-RCI threshold (log CN-RCI ≥ 0) used in LncATLAS to define the two classes.

**Figure 2:**
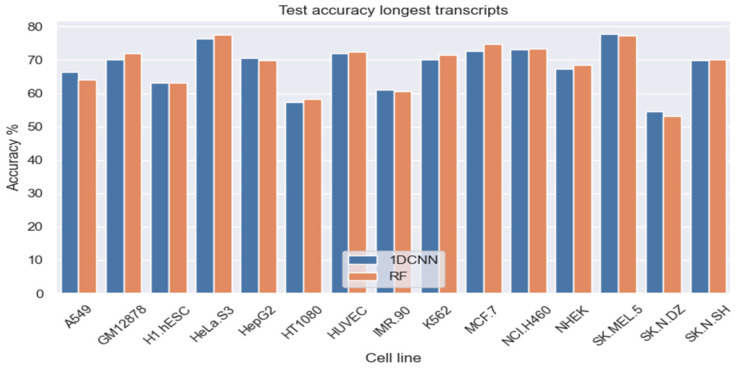
Test accuracy across 14 cell lines for lncRNA subcellular localization using RF (4096 features) and 1DCNN (512 latent features). Test set is based on the longest transcript for each lncRNA using the LncATLAS dataset.

Subsequently, we trained separate models for each of the 15 cell lines and tested the models using test data from the given cell line using our (6,2) inexact *q*-mer model. Based on observations from the earlier experiments with four cell lines, we only considered two classifiers in this more expansive evaluation, namely (a) 512 latent features using 1D-CNN and (b) all 4096 features using RF classifiers. Figure 2 shows the overall accuracy for the two classification models (1DCNN and RF) across the 15 cell lines, using the longest transcript for each gene. The average overall accuracy across the 15 cell lines was 68.14% for 1D-CNN (with 512 latent features) and 68.45% for RF (with all 4096 features).

### 3.2. Comparison with Published Benchmark Methods

In [61], the authors evaluated the effect of middle exclusion. They built the lncRNA subcellular localization dataset using canonical sequences of the genes. They tested the Random Forest (RF), gradient boost machine (GBM), support vector machine (SVM), and light gradient boost machine (LightGBM). They found that, when using unfiltered lncRNA data, the prediction accuracies of lncRNA subcellular localization were around 60%. They observed that predicting lncRNA subcellular localization from nucleotide sequences presents more complexity than currently acknowledged. We tested our methods with the lncRNA dataset used in [61]. We tested RF with all 4096 6-mers and 1DCNN with 512 latent features extracted from the 4096 features. The results are shown in Table 6. Using RF, 6-mer with one mismatch resulted in an accuracy of 62.29% ± 1.59 and an AUROC of 66.9 ± 2.27, which is slightly better than RF with zero mismatches on the benchmark dataset. Using 1DCNN, 6-mer with three mismatches, we obtained 63.57% ± 2.60 accuracy and an AUROC of 69. 11 ± 2.44. Table 6 shows the comparative performance of our proposed inexact *q*-mer approach, with popular methods without middle exclusion. The table shows that our proposed *(q,k)*-mismatch model provided an overall superior performance in lncRNA subcellular localization.

### 3.3. Is LncRNA Subcellular Localization Cell-Specific?

Using the CN-RCI values per gene per cell line provided by lncATLAS, we also explored the question of whether lncRNA subcellular localization is cell-type-specific or independent of the given cell type. To address this question, we considered three approaches: (1) correlation-based analysis, (2) cross-cell validation using our machine learning model, and (3) analysis of *switching lncRNAs* (also called shuttling lncRNAs).

First, we considered the possible correlation between the cell lines. We observed significant correlation between some pairs of cell lines, with some pairs having a correlation coefficient of over 0.8, for instance, 0.88 (HUVEC, IMR.90), 0.83 (SK.N.SH, HT1080), and 0.81 (MCF.7, HepG2). The cell lines IMR.90, SK.N.SH, and HUVEC were, overall, the most correlated with other cell lines, while the cell line H1.hESC (for human embryonic stem cell) appeared to be an outlier, with relatively low correlation with other cell lines (e.g., 0.3 (SK.MEL.5, H1.hESC)). A similar observation on H1.hESC was also made in [25]. Supplementary Figure S1 shows the detailed Pearson correlation coefficient between the 15 cell lines using the CN-RCI values. The significant correlation between certain pairs of cell lines implies that the CN-RCI values from one cell line could provide us with some information about some other cell lines, indicating that the CN-RCI values are not completely independent.

We then investigated potential similarities or differences between the different cell lines using results from the machine learning models. Using the same general setting for the previous experiments (threshold 0, (6,2)-inexact *q*-mers, MLP auto encoder (AE), and 1D-CNN for classification using 512 autoencoder latent features), we trained lncRNA localization models using each cell line and then tested on every cell line using the trained model. The expectation is that, if lncRNA subcellular localization is cell-line-specific, the highest performance accuracy will be observed along the left diagonal; otherwise, some off-diagonal elements will be significantly higher for some cell lines.

**Figure 3:**
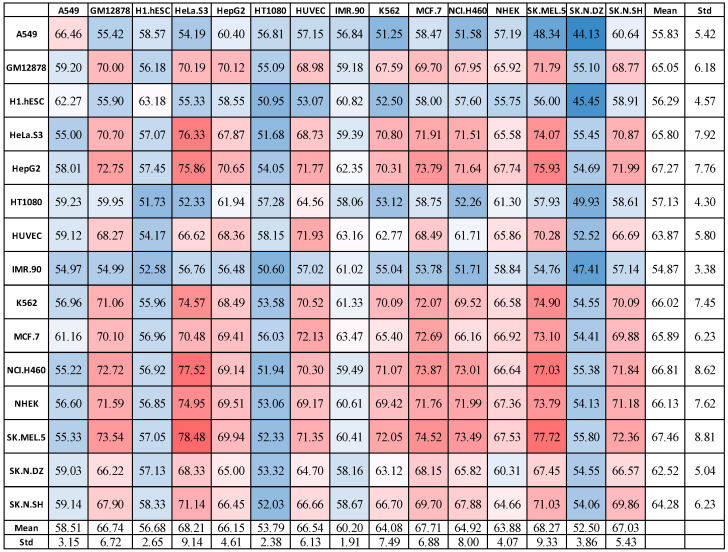
Results for train on one, test on all using (6,2)-mismatch model on 1DCNN with 512 latent features. In a red-white-blue color scale, red denotes high accuracy (dark red is with the highest score), white denotes accuracy close to the average, and blue represents low accuracy (dark blue denotes the lowest score).

Figure 3 shows the results, indicating performance for training on 1 cell line (the row) and testing on each of the 15 different cell lines (the columns). Interestingly, we found that, in almost every case, there were other cell lines that achieved higher accuracy than the original cell line (that is, many off-diagonal elements were higher than the diagonal elements). That is, a model trained on a given cell line could predict some other cell lines better than the original cell line it was trained on. For example, the NHEK-trained model scored 67.36% when tested on NHEK, but 74.95% overall on HeLa.S3, and 73.79 on SK.MEL.5. Similarly, a model trained on SK.N.DZ predicts most other models more accurately (on average) compared to predicting on SK.N.DZ.

Expectedly, training with the less-correlated cell lines, such as H1.hESC, led to reduced performance. Similar results were obtained using MLP and RF models. Overall, the results indicate that there are some clusters of cell lines wherein lncRNA localization in one is predictive for the others. This has a significant implication, as it suggests that it might be possible to develop a generalized model that can work well on most cell lines using data from only a few cell lines. This is more in line with the observation in [60], where the authors suggested that lncRNA localization may not be cell-specific.

To further investigate this issue of cell line specificity, we conducted an analysis of lncRNA localization distribution across cell lines using our training dataset. (Given that the H1.hESC cell line is an outlier, we did not include it in this analysis). For each cell line, we used a class threshold of log(CN-RCI) = 0, that is, an lncRNA is classified as nuclear if log(CN-RCI) < 0 and cytoplasmic if log(CN-RCI) >= 0. We set the label for nuclear lncRNA to 0 and for cytoplasmic lncRNA to 1. For each lncRNA, we counted the number of cell lines where it occurred in each of the two classes across all cell lines. We denote these counts as *C* and *N* for cytoplasmic and nuclear, respectively. We then define a switching lncRNA (also called switching gene) as one with C>1, N>1,  ≤1. Thus, a switching lncRNA will occur in the nuclear and cytoplasmic regions in at least two cell lines, respectively, and the number of cell lines with each compartment will be about the same across all the cell lines. Table 7 shows some examples of switching genes in our dataset. Column “A549” to column “SK.N.SH” show the 14 cell lines used for this analysis. Each row corresponds to an lncRNA gene. Each element in the table denotes the observed localization (label) of the lncRNA in the given cell line. An empty cell denotes when there is no available CN-RCI for the given lncRNA in the corresponding cell line. Columns “Cyto (C)” and “Nuclear (N)” record the number of cell lines where the lncRNA had cytoplasmic or nuclear localization, respectively. The column “Cell_count” = C + N is the total number of cell lines which have a CN-RCI value for the lncRNA. “C-N” denotes the difference between the number of cytoplasmic and nuclear localizations. As Table 7 shows, genes ENSG00000264207, ENSG00000248049, and ENSG00000117242 each appear in 13 cell lines and exhibit varying localization patterns. Using this method, we identified 185 switching genes. More detailed information on switching genes for our entire dataset can be found in Supplementary Table S2.

We conducted an in-depth analysis of these identified switching genes to gain further insights into this specific group of lncRNAs. We used three bioinformatics resources, namely, DAVID [64,65] (https://david.ncifcrf.gov/tools.jsp, accessed on 14 October 2024), a functional annotation tool, GeneCards (https://www.genecards.org/, accessed on 14 October 2024), a human gene database, and cncRNAdb [66], a manually curated resource of bifunctional RNAs. We queried the cncRNAdb and found that 12 of the 185 switching genes were identified as bifunctional lncRNAs (see Table 8). Bifunctional lncRNAs tend to appear in multiple localizations in a cell. We found that DAVID annotated 11 of the 185 switching genes. Five of these eleven, namely, CTBP1-DT, GNAS-AS1, OIP5-AS1, RHPN1-AS1, and SNHG7, were marked by GeneCards as being localized in multiple subcellular regions, such as nucleus and cytoskeleton or cytosol (see Table 9).

Overall, while lncRNA localization may not be cell-line-specific in general, the notion of switching genes and the demonstration of specific examples highlight the challenge of accurately predicting lncRNA localization via computational methods.

## 4. Conclusions and Discussion

LncRNAs can exist in different regions of the cell and show some crucial biological functions that may relate to diseases. Therefore, understanding their subcellular localization becomes an urgent task. However, compared to the vast number of lncRNAs, only a relatively small proportion have an experimentally validated annotation of their subcellular localization. It is possible to predict possible lncRNA subcellular localization using computational methods based on information from the few lncRNAs with known annotations or based on other related data, such as the cytoplasmic/nuclear relative concentration index (CN-RCI) values. The CN-RCI value was considered as a key signal that is associated with lncRNA subcellular localization [19]. But, there are no clear criteria to define the thresholds for different localizations. In this work, we considered lncRNA localization as a binary classification problem, namely, nuclear vs. cytoplasmic localization, and explored the use of deep learning methods to study certain problems in lncRNA localization. Further, gene mutations may affect the gene function and have been associated with certain genetic diseases. Here, we account for such mutations and other potential errors in the lncRNA sequence using the notion of inexact *q*-mers in our computational analysis of lncRNA localization. Our results showed that using the inexact *q*-mer (*q* = 6) profile (based on mismatches) can improve the prediction performance.

We further performed computational analysis using data from the lncATLAS and GENCODE databases to address the question of whether lncRNA localization is cell-line-specific. Our results showed that, in general, localization is not cell-line-specific. Thus, it may be possible to develop a general machine learning model that could work for most cell lines. However, our analysis also identified a category of lncRNAs that tend to switch between nuclear localization and cytoplasmic localization. That is, in some cell lines, they will appear to be more nuclear-leaning, while in some other cell lines, they are found to be more cytoplasmic-leaning. These switching lncRNAs make the automated prediction of lncRNA localization much more difficult.

We acknowledge some potential limitations in this work. First, the research relied primarily on CN-RCI values from lncATLAS. There are no specific criteria to define an appropriate threshold for nuclear and cytoplasmic localization using this data. The existing work that used this dataset set the threshold arbitrarily. More work needs to be completed to determine the best way to define the localization threshold(s) when using the CN-RCI values. Second, while the results on LncATLAS were comparable with the APEX-Seq dataset for mRNA transcripts, the results for LncRNA localization were worse when we trained our models on the LncATLAS dataset and tested on the APEX-Seq dataset or on the Ribosome dataset. This was particularly the case for AUC values (for APEX-Seq) and accuracy (for the Ribosome dataset). We speculate that this disparity in performance may be due to possible differences in the annotation or definition of the class labels between the datasets, for example, the logFC threshold used for the APEX-Seq data and the log (CN-RCI) threshold used for LncATLAS. This calls for further investigation, which could lead to new ways to improve the performance of the proposed method. Third, the datasets are unbalanced. With unbalanced datasets, a model may perform well at predicting the majority classes while doing poorly on the minority classes. We used SMOTE as the basic mechanism to handle imbalance in this work. More specific attention to this data imbalance problem could improve the results further. Furthermore, with the potential exponential increase in the feature space as *q* increases, computational challenges abound, both with respect to time and space. How to select the features is another challenge. These issues make a case for potential future directions building on the work described in this exploration.

## 5. Materials and Methods

**Figure 4:**
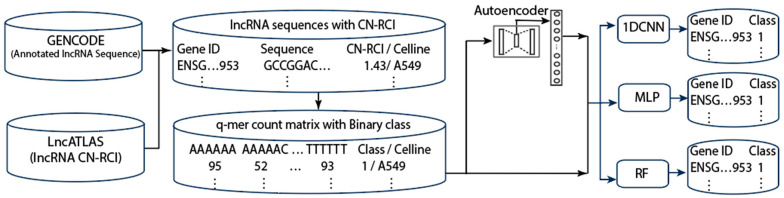
Workflow for the proposed method.

Figure 4 shows the workflow for our general methodology. We first downloaded the lncRNA sequence data from GENCODE (https://www.gencodegenes.org/human/, release V42, accessed on 14 October 2024)) and CN-RCI annotation data from lncATLAS [19]. We combined the sequence data and annotation data according to the gene’s GENCODE (Ensembl) ID. We then computed the frequency table for exact *q*-mers and inexact *q*-mers with *k* mismatches (*k* is from 0 to 3) in the lncRNA sequences. We used all 4096 features (with *q* = 6) to complete the classification. The feature space spanned by the *q*-mer increases exponentially with *q.* Further, high-dimensional features could lead to model overfitting when there are limited training data. These represent further computational challenges. To reduce feature dimension, we applied autoencoders [67] to extract 512 latent features and used the latent features to perform lncRNA localization prediction. We partitioned our data into training and test sets using a ratio of 4:1. We used the Synthetic Minority Oversampling Technique SMOTE [62] to handle the data imbalance while also investigating the effect of applying class weight when training the models. We conducted 5-fold cross validation twice on the training set and reported the average performance with the standard deviation.

### 5.1. Dataset

**Figure 5:**
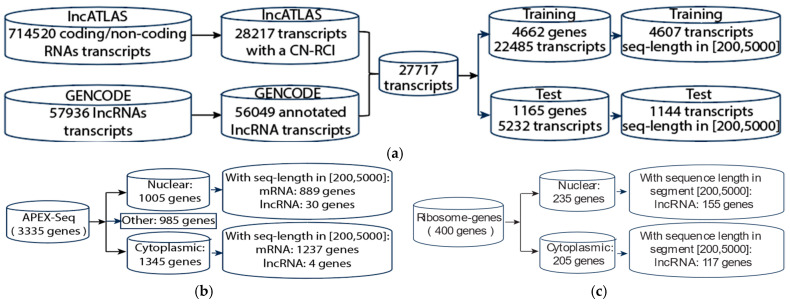
Building training set and test set from lncATLAS and GENCODE (**a**), APEX-Seq (**b**), and Ribosome (**c**) datasets.

In this study, we evaluated our methods on different types of localization datasets, namely, lncATLAS [19], APEX-Seq [55], and lncRNAs from ribosome profiling [63]. Figure 5a shows how we built our training and test sets based on lncATLAS. First, we downloaded localization data from lncATLAS [19], a database of lncRNA localization based on human RNA-seq data. It defines the localization using the relative concentration index (RCI), a log_2_-transformed ratio of FPKM (fragments per kilobase per million mapped) in cytoplasm and nucleus (CN-RCI). There are several attributes for the dataset: gene ensemble ID, cell line, CN-RCI value, gene name, and biotype (coding or non-coding). The dataset contains 714,520 entries of coding and non-coding RNAs. We screened out the coding RNA and kept the entries with non-NaN CN-RCI values. This resulted in 28,217 non-coding entries (lncRNAs) in 15 cell lines. Some lncRNAs had multiple CN-RCI values because they appeared in different cell lines. We also downloaded lncRNA sequence data and annotation files (.gff file) from GENCODE. The GENCODE dataset contained 57,936 lncRNA transcripts, including duplicated genes and duplicate transcripts for some genes. We retained 56,049 annotated transcripts where both gene_ biotype and transcript_ biotype are lncRNA.

Finally, we combined these two datasets according to the gene Ensembl ID and retained the transcripts which had at least one CN-RCI value for the cell lines and removed the genes without sequences in GENCODE. We called the resulting data the “all transcripts” dataset since it contained all transcripts for a given gene. We split the dataset into a training set and test set with a partition ratio of 4:1. To prevent possible data leakage between training and test sets, partitioning into training and test sets was performed in such a way that all transcripts for a given lncRNA were always placed in the same partition. We obtained 4662 genes and 22,485 transcripts in the training set and 1165 genes and 5232 transcripts in the test set (some genes had multiple transcripts). The length of the lncRNA transcripts in the training set varied from 72 to 205,000. To reduce the computation and ensure proper representation, we retained the genes with a sequence length of between 200 and 5000 in the training set. This accounted for 95% of the available data. We retained the longest transcript for each gene and removed duplicated genes. Finally, we obtained 4607 genes (one transcript per gene) in the training set (see Supplementary Table S3). Similarly, we obtained 1144 genes (also one transcript per gene) in the test set (see Supplementary Table S4). For a given cell line, some genes may or may not have a transcript with an RCI value. Thus, in our dataset, the total number of genes could vary between cell lines.

Similarly, we extracted mRNA data from GENCODE and lncATLAS [19] (see Supplementary Tables S5 and S6). We also tested on the canonical sequences dataset used in [61] (see Supplementary Tables S7 and S8).

We also tested our method on the APEX-Seq dataset [55]. APEX-Seq maps RNA locations by tagging nearby RNAs with biotin using an APEX2 enzyme in HEK293Tcells. They used H_2_O_2_ to activate the enzyme. By comparing samples treated with and without H_2_O_2_, they identified RNAs enriched in the cell component. They measured the comparison with log_2_ fold change. RNA with a log_2_ fold change (logFC) ≥ 0.75 is considered enriched in the component. In their study, they examined 8 components, namely, nucleus, nucleolus, lamina, nuclear pore, cytosol, endoplasmic reticulum (ER) membrane, ER_lumen, and outer mitochondrial membrane (OMM), for 3335 genes. Some of genes were enriched in multi-components according to the threshold. In this study, we consider only two classes, nucleus (including nucleus, nucleolus, and lamina) and cytoplasmic (including cytosol, ERM, ER_lumen, and OMM). We exclude nuclear pore because it serves as a dynamic gateway between the cytoplasm and the nucleus. With the genes they provided in their paper, we selected genes based on a log_2_ fold change threshold of 0.75 to assign class 0 for genes enriched in at least one nuclear localization and class 1 for genes enriched in at least one cytoplasmic localization. Then, we removed the genes with two classes. We then obtained the genes’ sequence from the dataset that we built previously for LncATLAS. This resulted in 2126 mRNA (1237 cytoplasmic and 889 nucleus) and 34 lncRNA (4 cytoplasmic and 30 nucleus) transcripts with a sequence length of between 200 and 5000 (see Figure 5b). Supplementary Tables S9 and S10 show the gene lists.

In 2016, Carlevaro-Fita et al. [63] applied polysome profiling coupled with spike-in normalized microarray analysis on the cytoplasmic and ribosome-associated population of stringently filtered lncRNAs in K562, a human cell line. They identified 440 genes and 637 transcripts localized in nucleus (292) and cytoplasm (345). Some genes have different transcripts localized in different locations. To be consistent with the data preparation for the lncATLAS dataset, we built a dataset based on the localization specified in [63]. We call this the Ribosome lncRNA dataset. The dataset uses the gene names from [63] but the longest transcripts of genes from our lncRNA dataset (based on lncATLAS). Here, the genes with lengths in (200, 5000) are retained. We finally obtain the Ribosome lncRNA dataset with 272 lncRNA transcripts, 155 nuclear and 117 cytoplasmic (see Figure 5c). The gene list is shown in Supplementary Table S11.

The localization distribution of lncRNA datasets from the lncATLAS [19], APEX-Seq [55], and Ribosome datasets [63] is shown in Table 10. The lncATLAS dataset contains 15 cell lines. We select cell line K562 to make the comparisons. The localization of the LncATLAS dataset was decided by the CN-RCI value of cell line K562. If CN-RCI ≥ 0, the location is cytoplasm; otherwise, it is nuclear. The APEX-Seq has a relatively small number of genes identified in nuclear and cytoplasmic localizations; however, it has a higher percentage sharing the same localization with lncRNAs in lncATLAS (21/34, or 61.8%) than those in the Ribosome lncRNA dataset (89/272 or 32.7%).

### 5.2. Feature Representation

LncRNA is transcribed from DNA and consists of nucleotide bases. These bases are adenine (A), guanine (G), uracil (U), and cytosine (C). The lncRNA sequence can be represented as *S* = *S*_1_*S*_2_…*S_i_…S_n_*, with *S_i_* ∈ {A, G, C, U}. Here, *n* is the length of the sequence and *S_i_* is the *i*th nucleotide base, 1 ≤ *i* ≤ n.

#### 5.2.1. *q*-mer Profile

A *q*-mer is a substring of a sequence with a length of *q.* For the case of lncRNA, each position in the *q*-mer consists of one of the four possible symbols: A, C, G, or U. Thus, for an lncRNA sequence, there are 4*^q^* (4 to power *q*) possible unique *q*-mers. Using the *q*-mers, we can construct the *q*-mer profile for a given lncRNA sequence, whereby the *q*-mer profile indicates the number of occurrences or frequency of each possible *q*-mer in the given sequence. Thus, we define the feature map (FM) of an lncRNA sequence as FM(*S*) = {*Q_i_*: *f_i_*, 1≤ *i* ≤ *N*}. Here, *S* is the sequence, *Q_i_* is the ith *q*-mer, *f_i_* is the corresponding feature value (that is, the frequency of the *q*-mer in S), and *N* is the number of possible unique *q*-mers in the sequence. For example, we can compute the 3-mer feature map for the sequence ***S* = *AGCUAGUA***. First, we find all the 3-mer combinations of *A, C, G*, and *U*. Then, we compute the frequency of each 3-mer. Finally, we obtain the feature map: FM(*S*) = {AAA:0, AAG:0, …, AGC:1, …, AGU:1, …, UUU:0}. The results are shown in the first row of Table 11.

#### 5.2.2. Inexact *q*-mer Profiles

In this work, we use the concept of inexact *q*-mer profile [26], with a focus on *q-*mers that allow for up to *k*-mismatch(es), also known as the (*q*,*k*)-mismatch kernel. This kernel incorporates the idea of mismatching in biological contexts [68,69] and can be used to capture potential changes due to mutations or sequence errors. For a given *q*-mer, we calculate the frequency of other matching *q*-mers, allowing for, at most, *k* mismatches. For this work, we set k≤⌊q/2⌋. Each matching *q*-mer is required to have the same length as the given *q*-mer. Therefore, the result of (*q*,*k*)-mismatch for a given *q*-mer, say *Q*, is a collection of *q*-mers, each having the same length as *Q* and with at least (*q*-*k*) bases that have an exact match with bases in *Q*. The Hamming distance is used to measure the mismatch.

Table 11 also shows the (3,1)-mismatch profile for the example sequence ***S = AGCUAGUA.*** This can be contrasted with the exact 3-mer profile, also shown in the table. Although we use a naive method to compute the (*q, k*)-mismatch feature map above, more efficient data structures such as suffix trees and suffix arrays [28] exist for faster computation of the *q*-mer profiles for both exact and inexact *q*-mers.

In this work, we consider the (*q*,*k*)-mismatch model, with *q* = 6 and (*k* = 0,1,2,3).

#### 5.2.3. Data Preprocessing

The (*q*,*k*)-mismatch will enhance the exact *q*-mer signals by increasing their frequencies. Once the feature maps have been extracted from the lncRNA sequences, both the exact 6 mer and inexact 6mer counts will be divided by the total counts of all exact 6mers in the sequences. This will reflect intrinsic relationship between exact and inexact 6-mers.

#### 5.2.4. Feature Encoding

The dimension of the feature map (or profile) is 4*^q^*, which grows exponentially with *q*. As the dimensionality of the feature map increases, it tends to become noisy, leading to a reduction in prediction accuracy and a higher risk of overfitting [70]. This is more problematic when there are limited data to cover all the possible unique *q*-mers. Additionally, as *q* increases, computational challenges arise due to the large number of features that must be handled. For instance, using 6-mers will result in 4096 features, which will result in a large feature space. Therefore, we need to reduce the size of the feature map. To address this issue, we applied an autoencoder [67] to obtain a latent feature space. We designed an autoencoder with a multi-layer perceptron (MLP) architecture. In encoder layers, we added two dense layers with 256 and 128 neurons followed by ReLU activation and batch normalization to normalize the activations to improve convergence during training. This was followed by dropout layer that randomly dropped 30% of neurons during training to prevent overfitting. Finally, the latent space was created with encoding dimension. The decoder mirrors the encoder, with 128 and 256 neurons for two denser layers, but reconstructs the data back to the original dimension. The final dense layer constrains the output between 0 and 1 (useful for binary data). In this study, we extract 512 latent features to analyze lncRNA subcellular localization.

#### 5.2.5. Predicting lncRNA Subcellular Localization

Few ncRNAs are expected to be completely nuclear or completely cytoplasmic at the steady state [71]. Thus, rather than one CN-RCI threshold, some researchers have argued for a consideration of a continuum between completely nuclear and completely cytoplasmic [71]. However, this leads to more complexity in the analysis. To simplify the analysis for our binary localization classification on the dataset, we follow recent work [58,59,61] and used a log2(CN-RCI value) of 0 as our class threshold. That is, if log2(CN-RCI) ≥ 0, we set the lncRNA class to 1 (i.e., cytoplasmic); otherwise, the class was set to 0 (i.e., nuclear). The class distribution for each cell line is shown in Supplementary Table S11.

To reduce the workload, we first chose to train models on four selected cell lines, HT1080, A549, NCI.H460, and SK.N.SH, which represented cell lines whose class distribution was near-balanced (HT1080), slightly skewed (A549), and highly skewed (NCI.H460, SK.N.SH). For each cell line, we built classification models using 1D-CNN, MLP, and RF on the 4096 6-mer features. We also tested 512 latent features, which were extracted from the 4096 features. To address the issue of data imbalance, we investigated both the use of SMOTE and the use of class weights during training. Overall, SMOTE showed a generally better performance over class weights. Thus, we applied SMOTE as our major approach against data imbalance.

### 5.3. Machine Learning Models

#### 5.3.1. 1D Convolutional Neural Network

**Figure 6:**
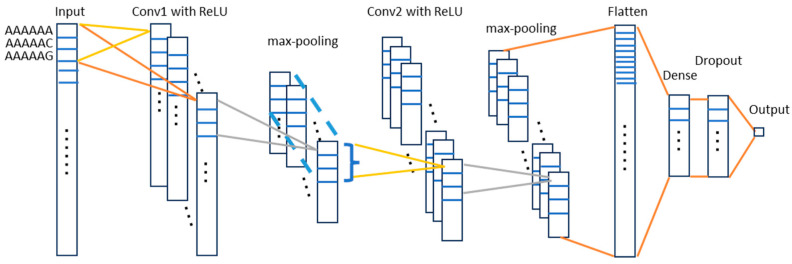
Proposed 1DCNN architecture for LncRNA sub-localization prediction. The grid on the bars denotes the frequency of the 6-mers. Dots on the bar denote omitted 6-mers. Dots out of the bar represent the sets of filters.

In this work, we trained a one-dimensional convolutional neural network (1D-CNN) model. The convolutional neural network (CNN) is a class of deep neural networks that employs a mathematical operation called convolution in at least one of its layers [70]. With convolution, a new feature map is constructed from the input features. 1D-CNN is designed to work on one-dimensional signals such as time series or sequential data. In this study, the processed lncRNA data possess two important characteristics: (1) each sample is represented by a fixed-length feature vector and (2) each *q*-mer is represented only by its frequency without incorporating its original sequence location. Given these conditions, applying a 1D-CNN model is technically feasible, although its performance may not be optimal. Therefore, we will also evaluate alternative models such as a Multilayer Perception (MLP) and Random Forest (RF) classifier to identify the most effective model. In this work, we built the 1D-CNN model with Keras [72]. Figure 6 shows the proposed architecture for the 1D-CNN model for classification. The input is the feature space: 4096 6-mer features or 512 latent feature space from the autoencoder. The first convolutional layer has 128 filters. Each filter has a size of 3 to capture local patterns, followed by ReLU activation function. The filter weights are initialized using He Normal initialization [73]. Each filter will produce a 1D feature map. The max-pooling layer downsamples the output of the filters by a factor of 3 (pool_size = 3), reducing dimensionality and computation. The output of the first max-pooling is then used as the input of the second convolutional layer. The structure of the second convolutional layer is similar to the first one but with 256 filters for deeper feature extraction. Each filter in the second convolutional layer will perform convolution across all 128 channels (results of first convolution layer, depth-wise operation). The second pooling layer reduces dimensionality by a factor of 3. The flattening layer converts the 1D feature map into a 1D vector, preparing it for the dense layers. A fully connected layer with 256 neurons processes the flattened features. ReLU activation is applied for non-linearity, while we randomly drop a fraction (30%) of neurons during training to prevent overfitting. A single neuron predicts the probability of the positive class (binary classification). The sigmoid activation maps the output to a range of between 0 and 1.

#### 5.3.2. Multilayer Perceptron

A Multilayer Perception (MLP) is a feed-forward artificial neural network that consists of a series of perceptrons stacked on top of each other. In this work, we built the MLP with Keras [72]. The model had three hidden layers; each layer had 128, 64, and 32 neurons with activation function “ReLU”, and the output layer had 1 neuron with activation function sigmoid to output a single value representing the probability of the positive class.

#### 5.3.3. Random Forest Classifier

Random Forest (RF) [74] is a supervised classification algorithm. It creates a forest with decision trees [75]. A decision tree partitions the space of input features into homogenous rectangular areas that correspond to an if–then rule over some input features. Given the training data with features and targets, RF will come up with a set of rules. RF uses these rules to make predictions on the test dataset. For a given sample in the dataset, each individual tree in the forest will produce a class prediction, and the class with the most votes will be the model’s output. We used an RF classifier from Scikit Learn [76] API using 100 trees in the forest.

### 5.4. Performance Evaluation

To evaluate the performance of the proposed machine learning classification methods in predicting lncRNA subcellular localization, we use overall accuracy (OA) and Matthew’s correlation coefficient (MCC) as the basic performance measures. This is computed as follows: OA = (TP + TN)/(TP + TN + FP + FN); *MCC* = (*TP* × *TN* − *FP* × *FN*)/$\sqrt{\left(TP+FP\right)\times \left(TP+FN\right)\times \left(TN+FP\right)\times \left(TN+FN\right)}$. Here, TP is the true positive, i.e., the number of positive samples that were predicted correctly. TN is the true negative, i.e., the number of negative samples that were correctly predicted. FP is the false positive, i.e., the number of negative samples that were incorrectly predict as positive. FN is the false negative, i.e., the number of positive samples that were predicted as negative. We also report precision (Pre) = TP/(TP + FP), recall (Rec) = TP/(TP + FN), F1-score = 2Pre × Rec)/(Pre + Rec), sensitivity (Sn, same as recall), specificity (Sp) = TN/(TN + FP), and Area Under the Receiver Operating Characteristic Curve (ROC-AUC) from prediction scores, which are computed using Scikit Learn [76].

## Figures and Tables

**Table 1:** Model review for LncRNA localization models that use *q*-mers as their primary feature. “—” denotes “not applicable”. (We indicated *q*-mer features; some models used additional features).

Methods	LncLocator [22]	iloc-LncRNA [23]	DeepLncRNA [24]	RNAlight [25]	TACOS [58]	Evaluation [61]	Our Model
Dataset	RNALocate	RNALocate	ENCODE project	GENCODE V30, APEX-Seq, LncATLAS	GENECODE, LncATLAS	GENECODE, LncATLAS	GENECODE, LncATLAS
Classes	5 classes	4 Classes	2 classes	2 classes	2 classes	2 classes	2 classes
Middle exclusion	—	—	Yes	Yes	Yes	Yes and No	No
Cell-line-specific	No	No	No	No	Yes	Yes	Yes
Feature	*q* mer (*q* = 4,5,6)	*q* mer (*q* = 8)	*q* mer (2,3,4,5)	*q* mer (*q* = 3,4,5),	*q* mer, (*q* = 1,2,3,4)	*q* mer, (*q* = 3,4,5)	Inexact *q* mer (*q* = 6)

**Table 2:** (a) Performance in predicting lncRNA subcellular localization with inexact 6-mers (6-mers with k mismatches) using 512 latent features. Average results for four cell lines (HT1080, A549, NCI.H460, and SK.N.SH). Results based on data from LncATLAS dataset. (b) Performance in subcellular localization prediction for LncRNAs using inexact q-mers. More detailed results for the (6,k)-mismatch model using 1DCNN with 512 latent features on LncRNA transcripts. Average results for four cell lines (HT1080, A549, NCI.H460, and SK.N.SH). Results based on data from LncATLAS dataset.

(**a**)															
		**0miss_oa**		**1miss_oa**	**2miss_oa**		**3miss_oa**		**0miss_auc**		**1miss_auc**		**2miss_auc**		**3miss_auc**
1DCNN		63.08 ± 5.86		67.89 ± 3.14	68.12 ± 3.08		67.19 ± 2.97		55.31 ± 3.85		60.44 ± 4.64		63.24 ± 4.51		61.64 ± 3.8
MLP		59.08 ± 5.35		62.2 ± 6.24	62.1 ± 5.07		63.37 ± 5.67		54.23 ± 2.96		56.83 ± 4.24		63.28 ± 4.52		61.48 ± 3.8
RF		63.15 ± 3.35		65.12 ± 2.7	66.27 ± 2.59		65.95 ± 3.01		59.58 ± 3.81		62.5 ± 3.7		63.15 ± 4.11		62.68 ± 4.18
(**b**)															
	**Sensitivity**		**Specificity**			**Precision**		**MCC**		**F1-Score**		**OA**		**AUC**	
0miss	0.53 ± 0.19		0.53 ± 0.19			0.49 ± 0.1		0.06 ± 0.06		0.5 ± 0.13		63.08 ± 5.86		55.31 ± 3.85	
1miss	0.55 ± 0.09		0.55 ± 0.09			0.52 ± 0.06		0.11 ± 0.05		0.52 ± 0.07		67.89 ± 3.14		60.44 ± 4.64	
2miss	0.57 ± 0.11		0.57 ± 0.11			0.55 ± 0.07		0.14 ± 0.08		0.55 ± 0.08		68.12 ± 3.08		63.24 ± 4.51	
3miss	0.56 ± 0.09		0.56 ± 0.09			0.56 ± 0.08		0.14 ± 0.06		0.55 ± 0.06		67.19 ± 2.97		61.64 ± 3.8	

**Table 3:** (a) Subcellular localization performance of the (6,2)-mismatch model using 512 latent features on mRNA transcripts. Average results for four cell lines (HT1080, A549, NCI.H460, and SK.N.SH). Results based on data from LncATLAS dataset. (b) More detailed results on subcellular localization performance for 1DCNN with 512 latent features using (6,k)-mismatch model on mRNA transcripts. Average results for four cell lines (HT1080, A549, NCI.H460, and SK.N.SH). Results based on data from LncATLAS dataset.

**(a)**															
	**0miss_oa**		**1miss_oa**		**2miss_oa**		**3miss_oa**		**0miss_auc**		**1miss_auc**		**2miss_auc**		**3miss_auc**
1DCNN	63.68 ± 1.9		66.07 ± 1.3		67.63 ± 1.37		52.3 ± 4.27		68.67 ± 2.0		71.57 ± 1.28		73.58 ± 1.33		49.96 ± 0.6
MLP	63.75 ± 1.63		65.54 ± 1.34		67.71 ± 1.1		54.7 ± 1.32		68.66 ± 1.48		71.12 ± 1.09		73.73 ± 1.07		50.14 ± 0.33
RF	64.22 ± 1.08		65.4 ± 1.02		67.12 ± 0.94		58.16 ± 1.55		68.77 ± 1.11		70.63 ± 1.12		72.88 ± 1.01		60.64 ± 1.68
(**b**)															
		**Sensitivity**		**Specificity**		**Precision**		**MCC**		**F1-Score**		**OA**		**AUC**	
0miss		0.64 ± 0.07		0.64 ± 0.07		0.64 ± 0.02		0.28 ± 0.03		0.63 ± 0.04		63.68 ± 1.9		68.67 ± 2.0	
1miss		0.66 ± 0.05		0.66 ± 0.05		0.66 ± 0.02		0.32 ± 0.02		0.66 ± 0.02		66.07 ± 1.3		71.57 ± 1.28	
2miss		0.68 ± 0.04		0.68 ± 0.04		0.68 ± 0.02		0.35 ± 0.02		0.67 ± 0.02		67.63 ± 1.37		73.58 ± 1.33	
3miss		0.5 ± 0.41		0.5 ± 0.41		0.4 ± 0.26		0.01 ± 0.04		0.35 ± 0.28		52.3 ± 4.27		49.96 ± 0.6	

**Table 4:** (a) Performance in predicting lncRNA subcellular localization with inexact 6-mers (6-mers with k mismatches) using 512 latent features on the APEX-Seq dataset (with threshold of log FC ≥ 0.75) using a trained model from the LncATLAS dataset (threshold of log (CN-RCI) ≥ 0). Average results for four cell lines (HT1080, A549, NCI.H460, and SK.N.SH). (b) Subcellular localization performance of the (6,k)-mismatch model using 512 latent features on mRNA transcripts using the APEX-Seq dataset.

(**a**)								
	**0miss_oa**	**1miss_oa**	**2miss_oa**	**3miss_oa**	**0miss_auc**	**1miss_auc**	**2miss_auc**	**3miss_auc**
1DCNN	68.38 ± 8.34	72.13 ± 6.95	68.75 ± 9.57	71.18 ± 6.86	48.25 ± 7.25	50.0 ± 8.0	46.5 ± 7.5	47.25 ± 5.75
MLP	59.71 ± 12.6	59.19 ± 12.37	54.12 ± 6.84	57.43 ± 10.92	47.5 ± 10.5	47.0 ± 7.75	43.5 ± 7.75	46.0 ± 8.5
RF	60.07 ± 6.32	66.54 ± 6.33	65.44 ± 6.38	66.76 ± 4.44	46.25 ± 8.0	45.75 ± 9.25	48.0 ± 7.0	48.0 ± 7.75
(**b**)								
	**0miss_oa**	**1miss_oa**	**2miss_oa**	**3miss_oa**	**0miss_auc**	**1miss_auc**	**2miss_auc**	**3miss_auc**
1DCNN	60.21 ± 2.17	59.99 ± 1.69	62.23 ± 1.98	52.58 ± 6.99	60.97 ± 4.1	61.42 ± 3.1	64.42 ± 2.75	51.02 ± 1.45
MLP	64.11 ± 0.65	65.15 ± 1.06	64.39 ± 1.71	63.76 ± 1.84	68.64 ± 1.87	69.65 ± 1.5	68.26 ± 2.25	69.18 ± 1.65
RF	62.42 ± 1.85	63.83 ± 1.7	63.59 ± 2.43	63.22 ± 1.44	65.16 ± 2.31	68.07 ± 2.15	67.64 ± 2.7	66.13 ± 1.88

**Table 5:** (a) Performance in predicting lncRNA subcellular localization with inexact 6-mers (6-mers with *k* mismatches) using all 4096 features (without the autoencoder). Data shown are validation results when using the Ribosome lncRNA dataset for both training and validation. (b) Performance in predicting lncRNA subcellular localization with inexact 6-mers (6-mers with *k* mismatches) using 4096 features on the Ribosome lncRNA dataset using a trained model from the LncATLAS dataset (threshold of log (CN-RCI) ≥ 0). Average results for four cell lines (HT1080, A549, NCI.H460, and SK.N.SH).

(**a**)								
	**0miss_oa**	**1miss_oa**	**2miss_oa**	**3miss_oa**	**0miss_auc**	**1miss_auc**	**2miss_auc**	**3miss_auc**
1DCNN	56.62 ± 4.5	60.64 ± 5.97	58.63 ± 5.41	59.18 ± 4.96	58.67 ± 7.59	64.0 ± 7.91	64.77 ± 8.05	63.62 ± 7.35
MLP	56.43 ± 6.19	57.16 ± 5.49	58.44 ± 6.29	59.37 ± 3.33	60.47 ± 6.56	62.07 ± 6.65	62.94 ± 6.0	59.12 ± 8.32
RF	55.68 ± 5.36	59.74 ± 6.36	58.82 ± 6.63	57.33 ± 7.78	57.18 ± 10.28	62.69 ± 6.73	60.86 ± 8.85	59.31 ± 9.05
(**b**)								
	**0miss_oa**	**1miss_oa**	**2miss_oa**	**3miss_oa**	**0miss_auc**	**1miss_auc**	**2miss_auc**	**3miss_auc**
1DCNN	57.38 ± 1.24	57.34 ± 1.51	57.02 ± 1.27	56.49 ± 1.17	58.75 ± 1.2	58.92 ± 1.59	56.52 ± 1.45	54.86 ± 1.42
MLP	53.74 ± 1.5	55.42 ± 0.96	54.28 ± 2.12	54.66 ± 2.39	55.42 ± 1.64	56.32 ± 1.98	53.97 ± 1.73	52.28 ± 1.84
RF	56.88 ± 1.71	56.66 ± 0.79	56.12 ± 1.48	55.37 ± 1.4	53.96 ± 2.74	56.61 ± 2.5	55.47 ± 1.95	54.99 ± 2.64

**Table 6:** Comparison with popular methods on the Benchmark dataset without middle exclusion. Results for the first five models are from the recent work in [56]. The last two columns are our methods. RF denotes our results with Random Forest using all 4096 features and our inexact (6,1)-mismatch model. 1DCNN denotes our results using 1DCNN with 512 latent features and our inexact (6,3)-mismatch model. “—” means no value reported.

Model:	RF	GBM	SVM	LightGBM	Benchmark (Balanced)	RF (Ours)	1DCNN (Ours)
Accuracy	61.8 ± 2	61.8 ± 1	62.9 ± 3	61 ± 2	60.9 ± 2	62.29 ± 1.59	63.57 ± 2.60
F1-score	59.1 ± 2	60.7 ± 2	60.6 ± 2	60 ± 2	60.0 ± 3	0.62 ± 0.02	0.64 ± 0.03
AUPRC	66.6 ± 4	65.2 ± 3	67.7 ± 3	66 ± 3	65.8 ± 3	—	—
MCC	0.239	0.235	0.261	0.227	0.219	0.25 ± 0.03	0.27 ± 0.05
AUROC	66.3 ± 2	66.2 ± 2	67.9 ± 3	66 ± 2	66.7 ± 1	66.9 ± 2.27	69.11 ± 2.44

**Table 7:** Example switching genes, showing the difficulty in developing computational methods for predicting lncRNA localization. See text for column headings. The numbers (0, 1) under each cell line denote the localization (nuclear and cytoplasmic, respectively). Blank cells imply no RCI values were available at the given gene for the cell line.

Gene_ID	A549	GM12878	HeLa.S3	HepG2	HT1080	HUVEC	IMR.90	K562	MCF.7	NCI.H460	NHEK	SK.MEL.5	SK.N.DZ	SK.N.SH	Cell Count	Nuclear (N)	Cyto (C)	C-N
ENSG00000264207	1	0	0	1	1	1	0	0	1	0		0	1	1	13	6	7	1
ENSG00000248049	1	1	0	0	1	0		0	0	1	1	0	1	0	13	7	6	−1
ENSG00000117242	0	1	0	1	0	1	1	1	0	1	0		0	1	13	6	7	1

**Table 8:** Bifunctional switching lncRNA genes identified by the proposed approach.

Gene	Description of Experimental Evidence (cncRNAdb)	Localization (GeneCards)
ENSG00000236719	The peptide was detected by LC-MS/MS in iPSC-derived cardiomyocyte.	lysosome/cytosol/endoplasmic reticulum/nucleus/mitochondrion/cytoskeleton/extracellular/plasma membrane
ENSG00000276814	The peptide was detected by LC-MS/MS in human cell lines (GM12878 cell).	no results
ENSG00000272168	The peptide was detected by heavy MRM in human cell lines (A549 cell; H1299 cell; HBE cell; Hep3B cell; MHCC97H cell; MHCCLM3 cell).	nucleus/golgi apparatus/cytosol/endosome/endoplasmic reticulum/mitochondrion/cytoskeleton/extracellular/plasma membrane
ENSG00000215386	The peptide was detected by LC-MS/MS in heart.	nucleus/cytosol/endoplasmic reticulum/mitochondrion/cytoskeleton/extracellular/plasma membrane
ENSG00000227486	The peptide was detected by LC-MS/MS in Hela cell.	novel transcript; no data for localization
ENSG00000117242	The peptide was detected by MRM in human cell lines (A549 cell; H1299 cell; HBE cell; Hep3B cell; MHCC97H cell; MHCCLM3 cell).	lysosome/cytosol/endoplasmic reticulum/nucleus/mitochondrion/cytoskeleton/extracellular
ENSG00000248690	The peptide was detected by LC-MS/MS in Hela cell.	lysosome/cytosol/endoplasmic reticulum/nucleus/mitochondrion/cytoskeleton/extracellular/plasma membrane
ENSG00000240875	The peptide was detected by LC-MS/MS in human cell lines (GM12878 cell).	nucleus
ENSG00000239219	The peptide was detected by MRM in human cell lines (A549 cell; H1299 cell; HBE cell; Hep3B cell; MHCC97H cell; MHCCLM3 cell).	no data for localization
ENSG00000234129	The peptide was detected by LC-MS/MS in anus.	no data for localization
ENSG00000261079	The peptide was detected by LC-MS/MS in salivary gland.	novel transcript; no data for localization
ENSG00000236013	The peptide was detected by in vitro translation assay.	no data for localization

**Table 9:** Switching genes annotated by DAVID.

Gene	Functional Annotation (DAVID)	Localization (GeneCards)
ENSG00000235831	BHLHE40 antisense RNA 1(BHLHE40-AS1)	nucleus
ENSG00000255568	BRWD1 antisense RNA 2(BRWD1-AS2)	no data for localization
ENSG00000196810	CTBP1 divergent transcript(CTBP1-DT)	cytosol/nucleus/mitochondrion/cytoskeleton/extracellular
ENSG00000175611	ERCC6L2 antisense RNA 1(ERCC6L2-AS1)	nucleus
ENSG00000235590	GNAS antisense RNA 1(GNAS-AS1)	nucleus/cytosol/endoplasmic reticulum/peroxisome/mitochondrion/cytoskeleton/extracellular/plasma membrane
ENSG00000272841	MAP3K4 antisense RNA 1(MAP3K4-AS1)	no data for localization
ENSG00000247556	OIP5 antisense RNA 1(OIP5-AS1)	nucleus/mitochondrion/cytoskeleton/extracellular/golgi apparatus/lysosome/cytosol/endosome/endoplasmic reticulum/plasma membrane
ENSG00000254389	RHPN1 antisense RNA 1 (head to head)(RHPN1-AS1)	lysosome/cytosol/nucleus/cytoskeleton/extracellular
ENSG00000278175	glioblastoma down-regulated RNA(GLIDR)	nucleus
ENSG00000221949	long intergenic non-protein coding RNA 1465(LINC01465)	no data for localization
ENSG00000233016	small nucleolar RNA host gene 7(SNHG7)	nucleus/cytoskeleton/extracellular/lysosome/cytosol/endosome/endoplasmic reticulum/peroxisome/mitochondrion/plasma membrane

**Table 10:** Distribution of lncRNA localization across different datasets. L denotes lncRNA dataset built from lncATLAS, R denotes Ribosome dataset, and A denotes the APEX-Seq dataset. “X and Y share” indicates the number of lncRNAs that both X and Y agree on for their localization.

Database	Nuclear	Cytoplasm	Total
LncATLAS [19]	808	539	1347
Ribosome [63]	155	117	272
APEX-Seq [55]	30	4	34
L and R share	54	35	89
L and A share	18	3	21
A and R share	0	0	0

**Table 11:** *q*-mer feature for S = AGCUAGUA showing both the exact 3-mer profile and the inexact (3,1)-mismatch profile (3 mer with one mismatch). “●●●” denotes omitted 3-mers in the feature map.

	AAA	●●●	AGC	●●●	AGU	●●●	GUA	●●●	UAG	●●●	UUU
Exact	0		1		1		1		1		0
1 mismatch	0		2		2		2		1		0

## Data Availability

The data presented in this study are available in this article. Source code and data for this paper can be found at https://github.com/bearstree/LncRNA-Subcellular-localization (accessed on 20 June 2024).

## Supplementary Materials

The following supporting information can be downloaded at: https://www.mdpi.com/article/10.3390/ncrna11040049/s1, Captions of the Supplementary files; Supplementary_Figure S1: lncRNA CN-RCI correlation; Supplementary_Table S1: 4096 feature with SMOTE and class_weight; Supplementary_Table S2: 185_switching_genes; Supplementary_Table S3: lncRNA_train_seq_with_RCI_longest_transcripts; Supplementary_Table S4: lncRNA_test_seq_with_RCI_longest_transcripts; Supplementary_Table S5: mRNA_train_seq_with_RCI_longest_transcripts; Supplementary_Table S6: mRNA_test_seq_with_RCI_longest_transcripts; Supplementary_Table S7: mean_RCI_positive.canonical; Supplementary_Table S8: mean_RCI_negative.canonical; Supplementary Table S9: APEX-Seq_lncRNA_transcripts; Supplementary Table S10: APEX-Seq_mcRNA_transcripts; Supplementary Table S11: ribosome_lncRNA_transcripts; Supplementary_Table S12: genes_distribution_per_cell_line.
