# Supplementary material for: LncRNA Subcellular Localization Across Diverse Cell Lines: An Exploration Using Deep Learning with Inexact q-mers
Source: Noncoding RNA. 2025 Jun 25;11(4):49. doi: 10.3390/ncrna11040049 (PMC12286058; doi:10.3390/ncrna11040049)
Supplement: Supplementary file 1 [file ncrna-11-00049-s001.zip › Supplementary_Figure S1_lncRNA_RCI correlation.pdf]

	A549	GM12878	H1.hESC	HeLa.S3	HepG2	HT1080	HUVEC	IMR.90	K562	MCF.7	NCI.H460	NHEK	SK.MEL.5	SK.N.DZ	SK.N.SH
**A549**	1.00	0.72	0.54	0.71	0.79	0.77	0.79	0.82	0.70	0.79	0.58	0.79	0.63	0.75	0.82
**GM12878**	0.72	1.00	0.42	0.71	0.81	0.71	0.78	0.71	0.83	0.72	0.58	0.70	0.67	0.74	0.68
**H1.hESC**	0.54	0.42	1.00	0.54	0.52	0.42	0.46	0.59	0.57	0.38	0.34	0.39	0.30	0.44	0.53
**HeLa.S3**	0.71	0.71	0.54	1.00	0.77	0.79	0.73	0.75	0.77	0.72	0.63	0.79	0.76	0.71	0.79
**HepG2**	0.79	0.81	0.52	0.77	1.00	0.80	0.86	0.83	0.81	0.81	0.61	0.76	0.72	0.76	0.81
**HT1080**	0.77	0.71	0.42	0.79	0.80	1.00	0.85	0.87	0.74	0.83	0.68	0.82	0.83	0.77	0.83
**HUVEC**	0.79	0.78	0.46	0.73	0.86	0.85	1.00	0.88	0.78	0.85	0.69	0.83	0.76	0.74	0.83
**IMR.90**	0.82	0.71	0.59	0.75	0.83	0.87	0.88	1.00	0.70	0.84	0.66	0.80	0.80	0.75	0.87
**K562**	0.70	0.83	0.57	0.77	0.81	0.74	0.78	0.70	1.00	0.73	0.56	0.67	0.70	0.80	0.67
**MCF.7**	0.79	0.72	0.38	0.72	0.81	0.83	0.85	0.84	0.73	1.00	0.65	0.79	0.78	0.74	0.83
**NCI.H460**	0.58	0.58	0.34	0.63	0.61	0.68	0.69	0.66	0.56	0.65	1.00	0.61	0.70	0.68	0.68
**NHEK**	0.79	0.70	0.39	0.79	0.76	0.82	0.83	0.80	0.67	0.79	0.61	1.00	0.74	0.70	0.83
**SK.MEL.5**	0.63	0.67	0.30	0.76	0.72	0.83	0.76	0.80	0.70	0.78	0.70	0.74	1.00	0.74	0.75
**SK.N.DZ**	0.75	0.74	0.44	0.71	0.76	0.77	0.74	0.75	0.80	0.74	0.68	0.70	0.74	1.00	0.71
**SK.N.SH**	0.82	0.68	0.53	0.79	0.81	0.83	0.83	0.87	0.67	0.83	0.68	0.83	0.75	0.71	1.00
