# Supplementary material for: LncRNA Subcellular Localization Across Diverse Cell Lines: An Exploration Using Deep Learning with Inexact q-mers
Source: Noncoding RNA. 2025 Jun 25;11(4):49. doi: 10.3390/ncrna11040049 (PMC12286058; doi:10.3390/ncrna11040049)
Supplement: Supplementary file 1 [file ncrna-11-00049-s001.zip › Supplementary_Table S1_4096 feature with SMOTE and class_weight.pdf]

Supplementary Table S1.a. Predicting lncRNA localization using 6-mers with mismatches, and SMOTE for class imbalance.

	0miss_oa	1miss_oa	2miss_oa	3miss_oa	0miss_auc	1miss_auc	2miss_auc	3miss_auc
**1DCNN**	68.60	68.34	**68.93**	68.72	0.63	0.65	0.64	0.64
**MLP**	65.83	66.14	66.02	**66.40**	0.62	0.63	0.62	0.61
**RF**	66.83	67.87	**68.20**	67.47	0.61	0.62	0.63	0.63

Supplementary Table S1.b. Predicting lncRNA localization using 6-mer with mismatches, and class weight during training for class imbalance.

	0miss_oa	1miss_oa	2miss_oa	3miss_oa	0miss_auc	1miss_auc	2miss_auc	3miss_auc
**1DCNN**	67.07	68.16	68.43	**68.47**	0.64	0.64	0.65	0.64
**MLP**	66.94	65.75	66.13	**67.64**	0.63	0.63	0.62	0.61
**RF**	67.37	68.18	68.13	**68.20**	0.61	0.63	0.63	0.62

Supplementary Table S1.c. Performance of 1DCNN model with 4096 features, with (6,k)-mismatch model on mRNA transcripts.

	Sensitivity	Specificity	Precision	MCC	F1-score	OA	AUC
**0miss**	0.64	0.64	0.65	0.29	0.64	64.74	0.70
**1miss**	**0.67**	**0.67**	0.67	0.34	0.67	67.57	**0.73**
**2miss**	**0.67**	**0.67**	0.67	0.34	0.67	67.62	**0.73**
**3miss**	**0.67**	**0.67**	**0.68**	**0.35**	0.67	**68.04**	**0.73**
